# Reliability and validity of handheld structured light scanners and a static stereophotogrammetry system in facial three-dimensional surface imaging

**DOI:** 10.1038/s41598-024-57370-x

**Published:** 2024-04-08

**Authors:** J. A. M. Schipper, B. J. Merema, M. H. J. Hollander, F. K. L. Spijkervet, P. U. Dijkstra, J. Jansma, R. H. Schepers, J. Kraeima

**Affiliations:** 1 Department of Oral and Maxillofacial Surgery, University Medical Center Groningen, University of Groningen, Groningen, The Netherlands; 2 Department of Rehabilitation Medicine, University Medical Center Groningen, University of Groningen, Groningen, The Netherlands; 3 Sirindhorn School of Prosthetics and Orthotics, Faculty of Medicine Siriraj Hospital, Mahidol University, 14 Arun Amarin Rd, Bangkok, 10700 Thailand

**Keywords:** Outcomes research, Three-dimensional imaging

## Abstract

Several new systems for three-dimensional (3D) surface imaging of the face have become available to assess changes following orthognathic or facial surgery. Before they can be implemented in practice, their reliability and validity must be established. Our aim, therefore, was to study the intra- and inter-system reliability and validity of 3dMD (stereophotogrammetry), Artec Eva and Artec Space Spider (both structured light scanners). Intra- and inter-system reliability, expressed in root mean square distance, was determined by scanning a mannequin’s head and the faces of healthy volunteers multiple times. Validity was determined by comparing the linear measurements of the scans with the known distances of a 3D printed model. Post-processing errors were also calculated. Intra-system reliability after scanning the mannequin’s head was best with the Artec Space Spider (0.04 mm Spider; 0.07 mm 3dMD; 0.08 mm Eva). The least difference in inter-system reliability after scanning the mannequin’s head was between the Artec Space Spider and Artec Eva. The best intra-system reliability after scanning human subjects was with the Artec Space Spider (0.15 mm Spider; 0.20 mm Eva; 0.23 mm 3dMD). The least difference in inter-system reliability after scanning human subjects was between the Artec Eva and Artec Space Spider. The most accurate linear measurement validity occurred with the Artec Space Spider. The post-processing error was 0.01 mm for all the systems. The Artec Space Spider is the most reliable and valid scanning system.

## Introduction

Three-dimensional (3D) surface imaging of the face is used for planning and evaluating orthognathic and facial surgery, for orthodontic diagnostics, and for research purposes^1^. The 3dMD (3dMD Inc., Atlanta, GA, USA) is the most widely used scanning system, using stereophotogrammetry, and its reliability and validity is reported in the literature^2,3^. However, the disadvantages are the system’s immobility and that certain areas of the face cannot be imaged completely due to the camera’s fixed orientation and focus point. An advantage is that the 3dMD’s modular system offers several set-up possibilities, enabling it to scan the whole body or parts of it. However, the modular system may lead to different measurement results due to having to apply a different number of pods and different lenses.

Several handheld scanners using structured light are gaining popularity and the results have been compared to those of the 3dMD system^4–6^. However, these structured light scanners use a sequence of pictures that form the 3D model instead of an individual 3D picture, whereby accuracy may be lost during post-processing. Structured light scanners acquire the surface of the face through continuous light emission, which undergoes distortions and deformations due to the irregularity of the surface^7^. Stereophotogrammetry, on the other hand, captures two or more images simultaneously from different angles. Before these new hand-held structured light scanners can be applied clinically, their reliability and validity need to be determined.

The Artec Eva (Artec Group Inc., Luxembourg, Luxembourg) has been used in multiple studies, showing poorer reliability then the 3dMD system^4^. According to the manufacturer’s specifications, the validity of the Artec Space Spider (Artec Group Inc., Luxembourg, Luxembourg) is better than the Eva system (www.artec3d.com). This manufacturer determined the systems’ validity by scanning “scale ball bars” with known distances between the balls. However, no study has compared the reliability and validity of the Artec Space Spider with the 3dMD system regarding scanning the facial region. The Artec Eva has a larger working distance than the Artec Space Spider, making the Eva system more useful for scanning larger areas such as the whole body. The manufacturer recommends the Artec Eva for scanning the face. The Artec Space Spider has a narrow field of view which leads to many small images being stitched together to create a 3D facial model. This procedure may introduce processing errors when scanning larger areas. Since more literature is available about the 3dMD, we selected two handheld structured light scanners with a different working distance and different reported validity to compare with the 3dMD. The aim of this study is therefore to determine the reliability and validity of 3D surface imaging systems, namely Artec Eva, Artec Space Spider and 3dMD, for the facial region by calculating both their intra-system and inter-system reliability, and the validity of the linear measurements.

## Material and methods

The study was approved by the medical ethics committee of the University of Groningen and University Medical Centre Groningen, the Netherlands (study number METC2021/476). The study protocol was in accordance with institutional guidelines and the Declaration of Helsinki. Written informed consent was obtained from all the participants prior to the study. The COSMIN taxonomy of measurement properties was used for the reliability and validity terminology and definitions (www.cosmin.nl). The subjects were healthy employees and students recruited from the department of oral and maxillofacial surgery at the University Medical Center Groningen (UMCG), hence we performed convenience sampling. An email was sent to the employees and students to ask if they would participate in this study. Volunteers were excluded from the study if there was severe facial deformity, excessive facial hair or if they had ever experienced epilepsy (because of the bright flashing light of the hand-held scanners). The volunteers were scanned with the static 3dMD, the hand-held Artec Eva and the Artec Space Spider systems. To ensure a natural head position, the volunteers were asked to look into the mirror, gently bite in maximum intercuspation, swallow before the scanning, relax their lips and to keep their eyes open^8^.

### Technical comparison of the scanning/scanner systems

The working distance of the Artec Eva is the smallest (0.2–0.3 m). The acquisition time and the processing time of the 3dMD are the smallest, 1.5 ms and immediate, respectively. The manufacturer reported that the Artec Space Spider (0.1 mm) has the highest accuracy, i.e. validity. The models produced by the Artec Space Spider capture the most details (Table 1).

**Table 1:** Overview of scanner systems.

	3dMDface system	Artec Eva	Artec Space Spider
3D model example			
Modality	Stereophotogrammetry	Structured light	Structured light
Handheld working distance	Not applicable	Yes (0.2–0.3 m)	Yes (0.4–1.0 m)
Acquisition time	1.5 ms	~ 20 s	~ 60 s
Processing time	Immediate reconstruction	Up to 10 min	Up to 30 min
Accuracy (reported by manufacturer)	0.2 mm	0.1 mm	0.05 mm
Resolution	0.5 mm	0.5 mm	0.1 mm
Cost	> 70,000 USD	> 15,000 USD	> 25,000 USD

### Data processing and analysis

**Figure 1:**
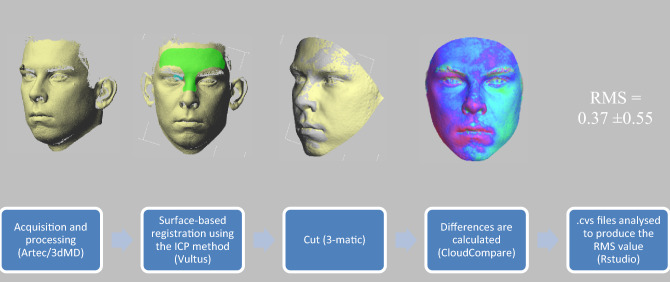
Processing steps (IC* p* = iterative closest point method; RMS = root mean square).

The data obtained after using the Artec Eva and Artec Space Spider were processed using the Artec Studio 14 Professional software (Artec Group, Luxembourg, Luxembourg) to acquire 3D models (Fig. 1). The 3dMD images were processed with the Vultus software (3dMD, Inc, Atlanta, GA, USA). As both Artec Eva and Artec Space Spider need additional steps to acquire a 3D model from the several frames, a standardized protocol from the manufacturer’s website^9^ was used for the processing in Artec Studio. The 3dMD Vultus software was utilized to match all the scan models by performing surface-based registration according to the iterative closest point method (this was performed for all scanning systems). After the images were manually aligned in the 3dMD Vultus software, a specific T-shaped area of the forehead (Fig. 1) and dorsum of the nose was marked on each scan as the registration surface. The quality of the registration was determined in the Vultus software and expressed as registration root mean square (RMS) error. The quality of the matching process was also determined based on a visual inspection. The scans were then cut with the 3-matic software (Materialise, Leuven, Belgium) by selecting planes formed by the landmarks tragus-forehead and tragus-menthon (Fig. 1). Then the scans were imported into CloudCompare (CloudCompare, version 2.11 alpha) and the cloud-to-mesh distance was computed with automatic octree level to produce signed distances (Fig. 1). In this mode, CloudCompare will simply search the nearest triangle in the reference mesh of each point of the compared cloud, i.e. the Euclidean distance. The RMS was calculated from these signed distances by RStudio (version 2021.09.2+382).

### Mannequin head intra-system reliability

**Figure 2:**
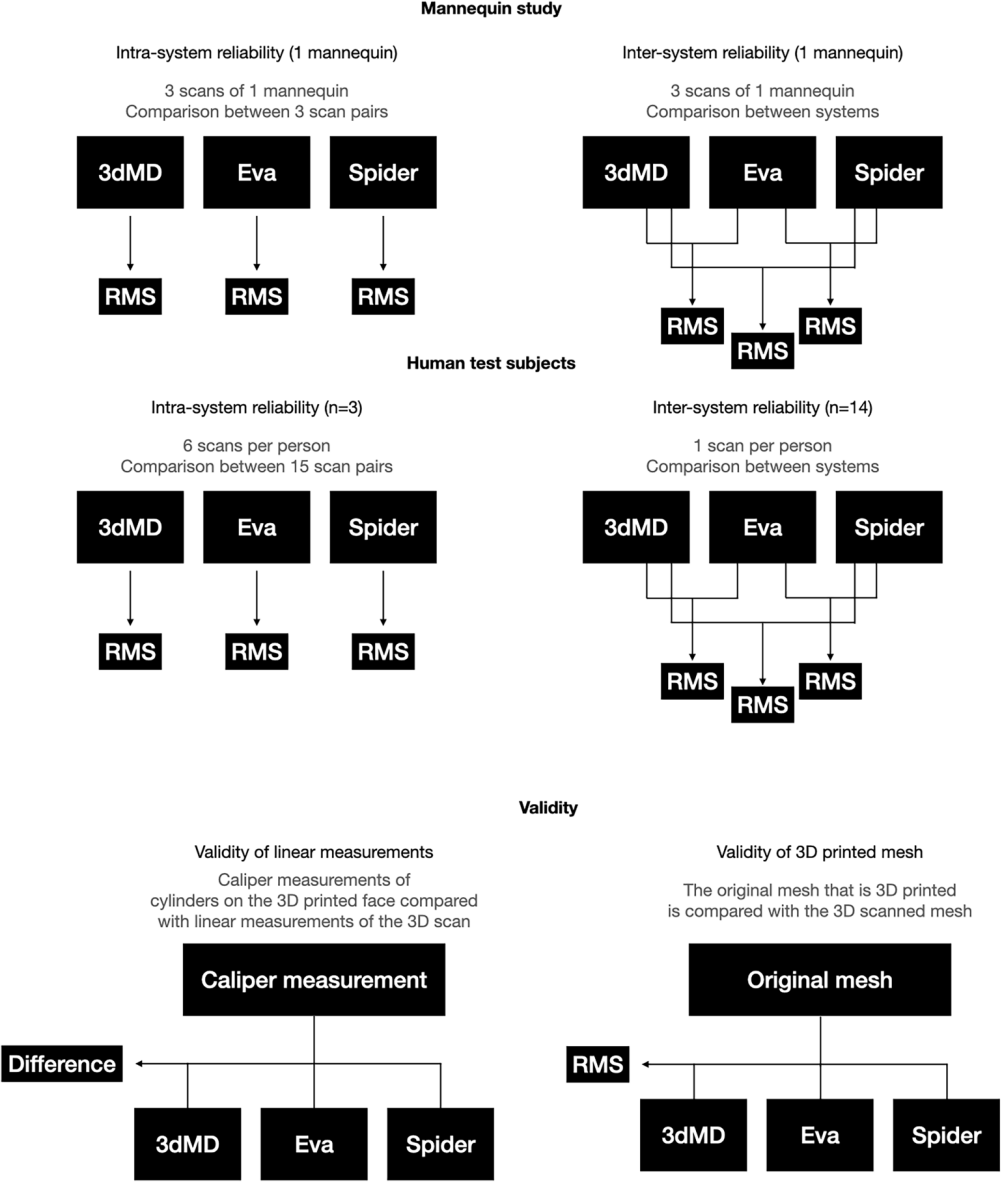
Study diagram (RMS = root mean square).

To check for the intra-system reliability of the scanners without interference of facial variability, a mannequin’s head was scanned three times with each system (Fig. 2). The RMS distances between the 3 scan pairs (1st scan vs. 2nd, 1st vs. 3rd, 2nd vs. 3rd) of each system were calculated.

### Mannequin head inter-system reliability

Distance maps were calculated for the mannequin head scans after being registered in the 3dMD Vultus software using histograms with identical colour-corresponding differences. The data acquired for the intra-system reliability were also used to analyse inter-system reliability. The RMS distances between the 1st scan of each system, between the 2nd scan of each system and the 3rd scan of each system, were calculated (1st scan 3dMD vs. 1st scan Artec Eva, 1st scan 3dMD vs. 1st scan Artec Spider, and 1st scan Artec Spider vs. 1st scan Artec Eva etc.).

### Intra-system reliability in 3 volunteers

To assess intra-system reliability on humans, 3 volunteers were scanned 6 times with each system (Fig. 2). The RMS distance was calculated for all 15 scan pairs of each system used on each volunteer (1st scan vs. 2nd, 1st vs. 3rd, 1st vs. 4th, 1st vs. 5th, 1st vs. 6th, 2nd vs. 3rd, 2nd vs. 4th, 2nd vs. 5th, 2nd vs. 6th, 3rd vs. 4th, 3rd vs. 5th, 3rd vs. 6th, 4th vs. 5th, 4th vs. 6th, 5th vs. 6th).

### Inter-system reliability in 16 volunteers

To assess inter-system reliability on humans, 16 volunteers were scanned with each imaging system once (Fig. 2). Each participant’s RMS distances were calculated and compared, Artec Eva versus 3dMD, Artec Space Spider versus 3dMD and Artec Eva versus Artec Space Spider. The combined participant RMS distance means were calculated for each of these pairs.

### Validity using a reverse engineered mannequin’s head as the gold standard

**Figure 3:**
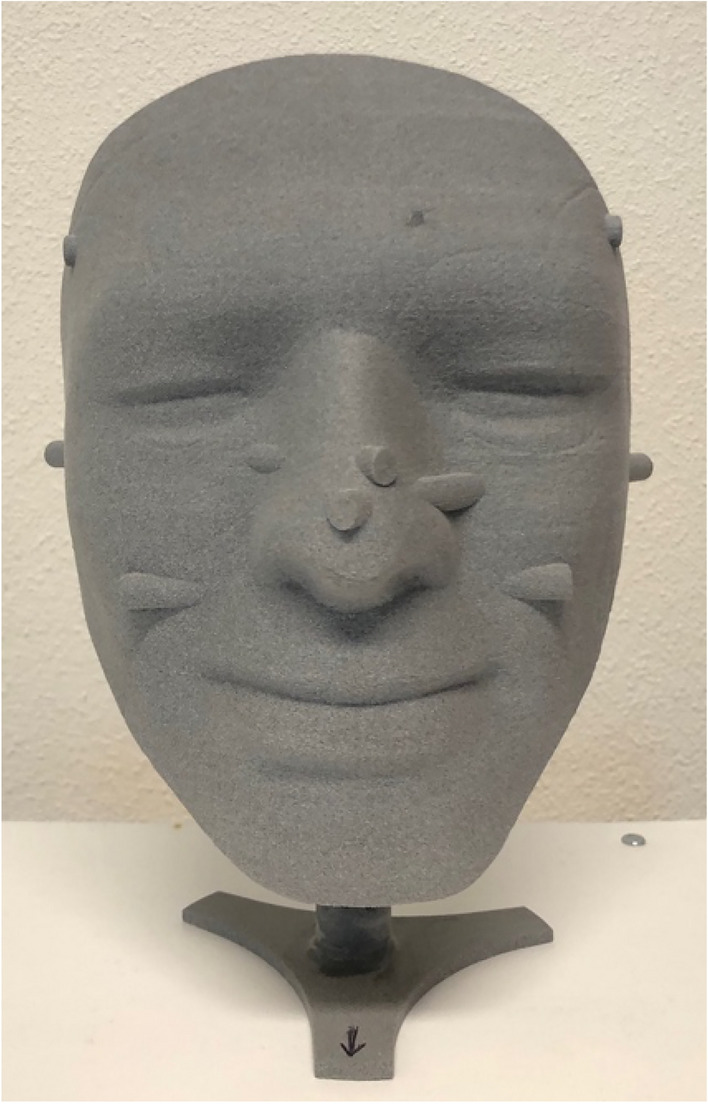
Reverse engineered 3D printed mannequin with known geometry and cylinders.

Since the precise surface dimensions of the mannequin’s face were unknown, we reverse engineered the mannequin’s face. The face was scanned using the Artec Space Spider and cylinders with known linear dimensions in multiple directions were modelled from the scan using the 3-matic software (Materialise, Leuven, Belgium). A 3D printer (PA12 material, EOS SLS printer, Oceanz, Ede, the Netherlands) was then used to print this reference model with known dimensions (Fig. 3). To compensate for potential errors in the 3D printing process, 2 observers took 3 repeated linear measurements with precise calipers (micrometres; with an accuracy of 0.01 mm) of the length of the cylinders of the 3D printed mannequin model to act as a gold standard. The 3D printed face was then scanned three times consecutively with the 3dMD, Artec Eva and Artec Space Spider systems. Linear measurements of these 3D scans were made from these cylinders with the 3-matic software (Materialise, Leuven, Belgium). The difference between the gold standard linear measurements and the 3D scanned measurements were used as validity measures. RMS distances were calculated for the 3dMD, Artec Eva and Artec Space Spider systems in relation to the reference model (the 3D printed mesh file).

### Post-processing error

Post-processing error was recorded by repeating the analysis process 5 times for one scan pair.

### Statistical analyses

All statistical analyses were performed using RStudio (version 2021.09.2+382). For the mannequin intra- and inter-system reliability descriptive statistics were provided, however no statistical testing was performed due to the limited sample size of 1 mannequin. Continuous variables were denoted as mean and standard deviation (SD). Normal distribution or skewness was assessed by visual inspection of the histograms and statistically tested with the Shapiro–Wilk test. Equality of variances was tested with the F-test. One-way repeated measures analysis of variance (ANOVA) was performed with a post hoc Tukey’s honestly significant difference test to check for the inter-system reliability after scanning human subjects. The Friedman test was performed as a nonparametric test when the conditions for parametric testing were not met.

### Informed consent

The human subject in the figures is the first author of this manuscript. The first author consents to publishing the figures.

## Results

### Mannequin head intra-system reliability

The Artec Space Spider had the best intra-system reliability (RMS 0.04 mm) whereas the reliability of scanning with the 3dMD and Artec Eva was similar (Table 2). The registration RMS error ranged between 0.02 and 0.04 mm.

**Table 2:** Intra-system reliability results after scanning a mannequin’s head 3 times with each system.

	3dMD (RMS in mm ± SD)	Artec Eva (RMS in mm ± SD)	Artec Space Spider (RMS in mm ± SD)
Mean difference ± SD	0.07 ± 0.01	0.08 ± 0.02	0.04 ± 0.01
Registration error	0.02	0.04	0.02

The RMS represents the mean difference between the 1st scan versus 2nd, 1st versus 3rd, 2nd versus 3rd.

*RMS* root mean square

### Mannequin head inter-system reliability

Visual inspection of the distance maps between the systems illustrated that 3dMD was less reliable than Artec Eva and Artec Space Spider with the main differences being in the following regions: under the nose, under the lip, and at the side of the face (Table 3). The distance maps showed more positive differences between the Artec systems and the 3dMD, especially at the sides of the face. Clinically this means that the Artec systems over-estimate or the 3dMD under-represents the true shape of the side of the face. In the Artec Eva vs. Artec Space Spider distance map, fine lines could be seen that were cuts made into the mannequin for a previous study. These cuts were captured better by the Artec Space Spider than the Artec Eva. The registration RMS error was 0.06 mm for Artec Eva versus Artec Space Spider, 0.08 mm for Artec Eva versus 3dMD, and 0.06 mm for Artec Space Spider versus 3dMD.

**Table 3:** Inter-system reliability results after scanning a mannequin’s head 3 times with each system.

	3dMD versus Artec Eva (RMS in mm ± SD)	3dMD versus Artec Space Spider (RMS in mm ± SD)	Artec Space Spider versus Artec Eva (RMS in mm ± SD)
Distance map			
1st scan	0.22 ± 0.20	0.17 ± 0.15	0.08 ± 0.15
2nd scan	0.19 ± 0.18	0.19 ± 0.19	0.15 ± 0.22
3rd scan	0.21 ± 0.17	0.16 ± 0.14	0.12 ± 0.16
Mean difference ± SD	0.21 ± 0.01	0.17 ± 0.03	0.12 ± 0.01
Registration error	0.07	0.06	0.06

The RMS represents the difference between the 3dMD versus Artec Eva, 3dMD versus Artec Space Spider, and Artec Space Spider versu Artec Eva system. Fine lines are visible on the head (arrow). These lines were cut into the mannequin for a previous study.

*RMS* root mean square

The best inter-system scanning reliability was between the Artec Space Spider and Artec Eva.

### Human subjects

Sixteen volunteers were scanned, but 14 scans were included in the statistical analyses since 2 volunteers had facial hair that produced errors on the surface models. Regarding the intra-system reliability measurements, 3 volunteers were randomly selected for extra scans. All 14 scans were used for the inter-system reliability assessment.

### Intra-system reliability in 3 volunteers

The Shapiro–Wilk test showed no significant deviation from the normal distribution. The F-test showed equal variances between the systems. Intra-system reproducibility did not differ significantly between the systems (repeated measures one-way ANOVA, *p* = 0.498). The mean RMS differences were smallest for Artec Space Spider (0.15 ± 0.02 mm) (Table 4). The registration RMS error was 0.07 mm for 3dMD, 0.09 mm for Artec Eva and 0.07 mm for Artec Space Spider.

**Table 4:** Intra-system reliability results after scanning 3 human subjects 6 times with each system.

	3dMD (RMS in mm)	Artec Eva (RMS in mm)	Artec Space Spider (RMS in mm)
Person 1 (15 scan pairs)	0.17 ± 0.03	0.26 ± 0.09	0.13 ± 0.05
Person 2 (15 scan pairs)	0.33 ± 0.12	0.16 ± 0.08	0.14 ± 0.03
Person 3 (15 scan pairs)	0.18 ± 0.03	0.18 ± 0.08	0.18 ± 0.04
Mean difference ± SD	0.23 ± 0.08	0.20 ± 0.05	0.15 ± 0.02
Registration error	0.10	0.06	0.06

RMS represents the mean difference between the scans for every person. The six scans of every person were compared in every possible combination which resulted in 15 scan pairs (1st scan vs. 2nd, 1st vs. 3rd, 1st vs. 4th, 1st vs. 5th, 1st vs. 6th, 2nd vs. 3rd, 2nd vs. 4th, 2nd vs. 5th, 2nd vs. 6th, 3rd vs. 4th, 3rd vs. 5th, 3rd vs. 6th, 4th vs. 5th, 4th vs. 6th, 5th vs. 6th). The intra-system reproducibility did not differ significantly between the systems (repeated measures one-way ANOVA, *p* = 0.498).

*RMS* root mean square

### Inter-system reliability in 14 volunteers

The Shapiro–Wilk test showed a deviation from the normal distribution for the 3dMD versus the Eva (*p* = 0.01), the 3dMD versus the Spider (*p* = 0.02) but not for the Spider versus the Eva system (*p* = 0.20), and so the Friedman test was used. The comparative inter-system scanning reliability differed significantly between the three systems, namely between the Artec Eva-3dMD, the Artec Space Spider-3dMD and the Artec Eva-Artec Space Spider (Friedman test; χ^2^ = 22.29; *p* < 0.01). Post-hoc Conover testing showed that the difference between the Spider vs the Eva system was less compared to the 3dMD versus the Eva system (*p* < 0.01; 0.26 ± 0.06 mm vs. 0.39 ± 0.11 mm, respectively), and the difference between the Spider versus the Eva system was less compared to the 3dMD versus the Spider system (*p* < 0.01; 0.26 ± 0.06 mm vs. 0.45 ± 0.11 mm, respectively) but there was no difference between the 3dMD versus the Eva system and the 3dMD versus the Spider system (*p* = 0.80; 0.39 ± 0.11 mm vs. 0.45 ± 0.11 mm, respectively). On comparing the 14 volunteers’ scanning results, the best inter-system reliability was between the Artec Eva and the Artec Space Spider systems (Table 5). The registration RMS error was 0.12 mm for the Artec Eva versus the Artec Space Spider system, 0.14 mm for the Artec Eva versus the 3dMD system, and 0.13 mm for the Artec Space Spider versus the 3dMD system.

**Table 5:** Inter-system reliability results after scanning 14 human subjects once with each system.

	3dMD versus Artec Eva (RMS in mm ± SD)	3dMD versus Artec Space Spider (RMS in mm ± SD)	Artec Space Spider versus Artec Eva (RMS in mm ± SD)
1	0.31 ± 0.31	0.33 ± 0.52	0.26 ± 0.24
2	0.69 ± 0.57	0.47 ± 0.53	0.21 ± 0.19
3	0.53 ± 0.64	0.41 ± 0.75	0.31 ± 0.36
4	0.27 ± 0.35	0.48 ± 0.95	0.24 ± 0.23
5	0.34 ± 0.25	0.38 ± 0.26	0.10 ± 0.14
6	0.35 ± 0.39	0.37 ± 0.55	0.21 ± 0.27
7	0.29 ± 0.75	0.34 ± 0.83	0.29 ± 0.25
8	0.32 ± 0.34	0.50 ± 0.88	0.24 ± 0.22
9	0.45 ± 0.54	0.75 ± 0.17	0.31 ± 0.27
10	0.42 ± 0.39	0.44 ± 0.54	0.26 ± 0.23
11	0.32 ± 0.33	0.40 ± 0.74	0.26 ± 0.24
12	0.45 ± 0.41	0.44 ± 0.74	0.30 ± 0.31
13	0.35 ± 0.35	0.34 ± 0.62	0.34 ± 0.32
14	0.35 ± 0.35	0.59 ± 1.00	0.34 ± 0.28
Mean difference ± SD	0.39 ± 0.11	0.45 ± 0.11	0.26 ± 0.06
Registration error	0.14	0.13	0.12

RMS: Root mean square. RMS represents the mean difference between the scans from the different systems. The Friedman test and post-hoc Conover testing showed significant differences between Spider versus Eva and 3dMD versus Eva (*p* < 0.01), Spider versus Eva and 3dMD versus Spider (*p* < 0.01), but no difference between 3dMD versus Eva and 3dMD versus Spider (*p* = 0.80).

### Validity using reverse engineered mannequin head as gold standard

Digitally performed linear measurements on the 3D scans compared to the gold standard showed that the Artec Space Spider had the best validity (Table 6). The Artec Eva had consistently higher measurements, while the 3dMD demonstrated consistently lower measurements. When comparing the 3D printed model to the three systems’ scans, the Artec Space Spider was also the most accurate in scanning the geometry, showing the lowest RMS difference between these meshes. The distance maps showed that the three systems’ scans mostly differed in the lower part of the face.

**Table 6:** Reverse engineered mannequin head with cylinders for gold standard use (RMS = root mean square).

	3dMD versus mannequin model	Artec Eva versus mannequin model	Artec Space Spider versus mannequin model
Distance maps			
Linear measurements error (deviation in mm ± SD)	0.30 ± 0.07	0.30 ± 0.10	0.03 ± 0.02
Mean difference (RMS in mm ± SD)	0.38 ± 0.06	0.36 ± 0.07	0.31 ± 0.03
Registration error	0.21	0.39	0.10

### Post-processing error

After repeating all the described processing steps five times for one scan pair, a standard deviation of 0.01 mm was found for all 3 scanning systems.

## Discussion

Three-dimensional surface imaging has proven to be a valid and reliable imaging modality for evaluating several surgical treatment results of the face. New handheld scanning systems have been released which advertise even higher accuracy than the previously widely used static systems. Reliability studies of these new scanners are needed before they can be applied safely in clinical practice and research.

Intra-system and inter-system reliability was established in this study by scanning both healthy volunteers and a mannequin’s head. We found that the Artec Space Spider gave better intra-system reliability results for both the volunteers and the phantom head. The Artec Space Spider captured more details, since small wrinkles and small protuberances of the face were visualized very well. The differences in reliability between the Artec Space Spider and Artec Eva were small whereas, compared to these handheld scanners, the 3dMD system introduced more inaccuracy as illustrated by the distance maps, especially under the nose, under the chin and at the side of the face. These differences could possibly be explained by the fixed orientation of the 3dMD system. The Artec handheld systems can scan in several angles around the face to capture iterative meshes that are stitched together to produce a complete 3D model. Therefore, the geometry underneath the nose or chin can be imaged more completely using handheld systems. Of note, though, is that due to the narrow field of view of the Artec Space Spider, many iterative meshes must be captured and stitched together to create a 3D model. This process can hypothetically introduce a processing error when scanning large areas. However, since the overall reliability and validity of the Artec Space Spider was high in our study after scanning the face, we consider the relevance of this error to be low.

Moreover, since the errors were all below 0.5 mm, the clinical relevance of these differences are very limited. Other properties such as ease-of-use or the time needed to generate a 3D model could dictate the choice for a specific system. However, when measuring volume by comparing larger surface scans of the face, these small differences add up to more inaccurate volume measurements. Therefore, we recommend using a system with the smallest errors, such as the Artec Space Spider, for pre- and post-surgery volume measurements.

In previous studies, 3dMD was used as a reference when comparing between scanners^6^. Yet, since the reliability of the other scanners tested in this study was better than 3dMD, we believe that 3dMD should not act as a gold standard anymore. We 3D printed a mannequin phantom head with cylinders. Since 3D printing introduces errors, we took linear measurements of the cylinders, in multiple directions on the mannequin’s head, to operationalize this as a gold standard. These measurements show that the Artec Space Spider is more valid in capturing the geometry of the face. Based on the linear measurements error, it seems that the Artec Eva overestimates the volume of the face and the 3dMD underestimates the volume of the face.

Most studies have analysed the reliability and validity of the 3dMD stereophotogrammetry system^2,3,10^. A recent study which scanned dental casts showed that the Artec Space Spider had high reliability, but low validity (0.4 mm)^11^. However, the authors attributed the error to the automatic reconstruction function of the software, a function we did not use. The Artec Space Spider gave one of the best validity measures compared to the Primescan, Trios, Pritiface and iPhone systems when used to scan nasal, orbital and auricular models that were manufactured using stereolithography^12^. The Artec Space Spider’s results were found to be most valid compared to Artec Eva, Vectra H1, Bellus and SNAP after scanning plaster statues with balls attached; the linear measurements were used for comparison purposes^13^. The reliability of scanning the peri-orbital region with the Artec Space Spider was excellent, at 0.1–0.2 cmm^14^.

In this study, we showed that all 3 scanners produce clinically acceptable results, with errors of less than 0.3 mm for intra-system reliability. Variation in facial expression is known to introduce an error when comparing multiple facial 3D surface scans, which explains the differences between the human volunteers and the phantom head^15^. The substantial difference between the phantom head and the faces’ RMS distances shows that the variability in facial expression introduces more error than the scanning systems themselves. This finding highlights that standardization of facial expression during scanning could be even more important than scanning system accuracy. Regarding clinical use, certain properties can differentiate their usefulness for specific applications. The Artec Space Spider is especially useful for acquiring scans of objects with complex geometry, since it showed the best validity and captured the most detail. Artec Eva and Artec Space Spider are mobile scanners, a property which makes them especially useful when the scanner needs to be used at multiple sites, such as the operating room or multiple clinical centres. In addition to this, the storage space needed for these mobile scanners is substantially less than the static 3dMD scanning system, which needs a designated room for its setup. However, training and experience is required for the mobile scanners to produce acceptable scan quality since slow and homogenous movements are required during the recording. The 3dMD obtains surface models with a single flash which reduces the risk of movement artefacts during scanning, and provides fast acquisition times.

One of the strengths of our study is that we included both intra- and inter-system reliability of scanning a mannequin’s head and human subjects. This facilitates proper assessment of the reliability of both a rigid object with the geometry of a face and the face of a human subject. We also included validity analysis of a 3D printed face and linear measurements of cylinders on this 3D print. However, the main limitation of our study is that the true geometry of the human face is not known which influences validity measures. It is therefore impossible to define a gold standard which can facilitate the assessment of the validity of capturing the true complex geometry of the face. We therefore also assessed a 3D printed face and compared this with the original printed mesh. However, as 3D printing also introduces a printing error, we also made linear measurements with calipers and compared these with the linear measurements of the 3D scans, consequently showing again that the Artec Space Spider’s results are the most valid. Other limitations are the study’s small sample size and that we did not perform a formal sample size calculation, but used convenience sampling based on sample sizes from the literature. Despite these limitations, the multiple analyses give consistent results: the Artec Space Spider scanning system is the most reliable and valid. Future research on the validity of new 3D surface imaging systems should therefore focus on establishing a proper gold standard for facial scanning.

We have proven that the three compared scanning systems are reliable and valid but conclude that the Artec Space Spider is the most reliable and valid. Other properties such as ease-of-use or mobility of the 3D surface imaging systems can dictate which system to choose in practice. Nonetheless, 3D surface imaging is a promising and radiation-free imaging modality that can be safely implemented to assess facial surgical outcomes during the follow-up.

## Data Availability

Data is available at the research databank of the department of Oral and Maxillofacial Surgery in the University Medical Centre of Groningen, the Netherlands. Data can be requested through the first author, J.A.M. Schipper (j.a.m.schipper@umcg.nl). It can also be requested through our department administration (+31 503613840; l.kempers@umcg.nl).
